# Roy Walford and the immunologic theory of aging

**DOI:** 10.1186/1742-4933-2-7

**Published:** 2005-04-25

**Authors:** Rita B Effros

**Affiliations:** 1 Department of Pathology and Laboratory Medicine, David Geffen School of Medicine at UCLA Los Angeles, CA 90024, USA

**Keywords:** Ageing, Immunity, Immunosenescence, Walford

## Abstract

Roy Walford died on April 27, 2004, at the age of 79. His contributions to gerontological research in such diverse areas as caloric restriction, genetics of lifespan, immunosenescence, DNA repair and replicative senescence were truly remarkable in their depth and innovation. Significantly, most of the areas that he pioneered during his illustrious research career remain the "hot" areas of current gerontological research. In this sense, he has achieved the most important type of immortality. His death was a major personal and professional loss to numerous scientists within the gerontological community. In launching this new journal on Immunity and Ageing, it is highly fitting, therefore, to remember him on the anniversary of his death by briefly reviewing the contributions of Roy Walford to this important facet of gerontology. Indeed, it was Roy who actually first coined the commonly used term "immunosenescence".

## Introduction

Dr. Roy Walford, M.D. Professor Emeritus of Pathology and Laboratory Medicine at UCLA, died on April 27, 2004 after a long battle with amyotrophic lateral sclerosis. He joined UCLA's faculty in 1954 and became Professor of Pathology in 1966. He is widely regarded as a pioneer in the study of the biology of aging. He and his colleagues have made major contributions to several areas of experimental gerontology including caloric restriction, immunological aging and the role of body temperature reduction in aging retardation. Early in his career he was also a leader in the study of histocompatibility systems. Last year a special issue of Experimental Gerontology was dedicated to Roy Walford. In that volume, the comments of several colleagues with whom Roy interacted in fundamental areas of immunology provided evidence of the admiration and respect with which he was viewed. For example, the eminent Nobel Laureate, Professor Jean Dausset, honored for his contributions to the field of human leukocyte antigen biology, which is central to immune function, described Roy as "brilliant..... original....lucid visionary of the future" [1]. Dr. Edwin Cooper expressed his gratitude for the novel contributions to immunosenescence derived from his interactions with Roy in the study of lower animals and phylogenetic analyses [2]. The collaboration between Roy and Dr. G.B. Ferrara in identifying the so-called "Merrit alloantigen system", now known as MHC Class II, were of fundamental importance in both transplant immunology and more recently, in understanding antigen presentation. Dr. Ferrara described Roy as "an extraordinary man: genius, scientist, philosopher, artist, predictor of the events of our time" [3]. This unusual foresight and intuition probably played a role in the fact, as stated by immunologist Dr. Richard Miller, that Roy was among the first "to note and promote the powers of modern immunological approaches as tools for the analysis of ageing" [4]. Thus, Roy has played a pivotal role in numerous and varied aspects of immunology and immunological ageing. His contributions to gerontological research were truly remarkable in their depth and innovation. Significantly, most of the areas that he pioneered during his illustrious research career remain the "hot" areas of current gerontological research. In this sense, he has achieved the most important type of immortality. His death was a major personal and professional loss to numerous scientists within the gerontological community.

On this first anniversary of Roy's death, it is highly fitting to remember him by briefly reviewing the contributions of Roy Walford to this important facet of gerontology, by this new journal devoted to Immunity & Ageing. Indeed, it was Roy who actually first coined the commonly used term "immunosenescence". In 1969, Roy Walford published his landmark book, "The Immunologic Theory of Aging" [5], which has now become a classic. This book, together with his 1961 National Institutes of Health grant on "The role of immune phenomena in the ageing process", which was continuously funded for nearly 40 years, formed the basis for many of today's ideas about immunological ageing. Briefly, Roy hypothesized that the normal process of ageing in man and in all animals is pathogenetically related to faulty immune processes. This notion has proven to be prophetic. Indeed, in humans, numerous clinical studies show significant correlations between specific immune functional traits and early mortality, irrespective of the cause of death [6,7]. Moreover, even in C. elegans, longevity is associated with increased resistance to bacteria [8], underscoring the evolutionarily-conserved link between immunity and lifespan.

## Replicative senescence within the immune system

Roy was a visionary, and had the ability, so rare among scientists, to see the forest, and not just the trees. Indeed, it was this sort of thinking that led him to identify a novel field of research as being of potential relevance to human ageing, namely, the role of the cellular program of replicative senescence within the immune system. It is this merging of two distinct fields of scientific research that exemplifies the highly creative aspects of Roy's thinking. Specifically, during the early 1980's, Roy made the astute observation that within the scientific community, there existed two major groups of researchers, namely, immunologists and cell biologists, whose views on an important aspect of cell behavior were mutually incompatible. Amazingly, for at least 7 years both disciplines developed in parallel with very little manifest cognizance of a major conflict of theoretical tenets [9].

A debate about replicative senescence arose with respect to replicative senescence and its potential role in the immune system. The well known studies of Hayflick [10] and Hayflick & Moorhead [11] had suggested that normal vertebrate diploid cells cultured in vitro invariably undergo a finite number of cell divisions. Although there are many theoretical explanations for this so-called "Hayflick Limit", and despite the fact that its potential role in the ageing process is still unresolved, the dogma itself has become a cornerstone of cell biologists' and gerontologists' thinking. In the same period when replicative senescence research was flourishing, it happened that a novel cytokine which promoted T cell proliferation in cell culture was discovered, an event that soon led to a flurry of immunology papers describing the seemingly unlimited growth of normal human T cells [12,13]. This apparent conflict between immunologists and cell biologists captured Roy's attention, and he was anxious to investigate whether or not human lymphocytes cultured in vitro were, in fact, restricted by the Hayflick Limit [9]. He reasoned that since immune responses to foreign pathogens require extensive proliferation, a limited replicative potential might have a significant impact on the efficient function of lymphocytes, particularly in the elderly.

To systematically analyze the proliferative potential of lymphocytes, human T cells derived from peripheral blood of healthy young adult donors were propagated in cell culture by repeated stimulation with antigen and continuous exposure to the T cell-specific growth factor, Interleukin-2 (IL-2). The mean number of cumulative population doublings, derived from hundreds of cell cultures analyzed, falls consistently between 25 and 40. This limited range has been reported by several different investigators who studied either bulk cultures or clonal populations, and it applies equally to the two subtypes of T cells (CD4+/helper and CD8+/cytotoxic) [14-16]. The few studies which analyzed cell populations in the rare immortal cultures derived from normal T cells did, in fact, find karyotypic abnormalities. Thus, it has now been firmly established that T cells are similar to other normal human somatic cells in that they invariably undergo replicative senescence in cell culture [17-19]. Moreover, these studies identified several senescence-associated alterations that are restricted to T cells, in addition to those that had already been documented in other cell types, such as increased cell cycle inhibitor proteins, reduced response to stress, telomere shortening and resistance to apoptosis [16,20-22].

## Roy Walford's legacy

Research over the past few decades has repeatedly confirmed the insightful predictions made by Roy Walford regarding the role of the immune system in various pathologies of aging. Indeed, in accord with Roy's original hypothesis on the role of immunosenescence in human ageing, there is accumulating evidence that many of the so-called "diseases of aging" are caused by dysregulated immune function and excessive inflammation [23]. For example, Alzheimer's disease patients show a correlation between mental function and T cell telomere length [24]. In addition, there is increasing evidence of T cell involvement in atherosclerosis [25]. Longitudinal studies on the elderly have also provided clinical validation of many of Roy's theoretical predictions. Analysis of the Swedish geriatric population over several decades, for example, has documented a series of immune characteristics that is associated with early all-cause mortality. This so-called "immune risk profile" includes high proportions of CD8 T cells that lack CD28 expression as well as poor T cell proliferative capacity [6,7], both of which are hallmarks of T cell replicative senescence.

The underlying mechanisms for many of the above clinical correlations remain to be determined, but the trajectory and agenda for a large body of immunogerontological research was established long ago, thanks to the original far-reaching predictions and hypotheses posed by Roy Walford.
